# LINCS L1000 dataset-based repositioning of CGP-60474 as a highly potent anti-endotoxemic agent

**DOI:** 10.1038/s41598-018-33039-0

**Published:** 2018-10-08

**Authors:** Hyun-Wook Han, Soojung Hahn, Hye Yun Jeong, Joo-Hyun Jee, Myoung-Ok Nam, Han Kyung Kim, Dong Hyeon Lee, So-Young Lee, Dong Kyu Choi, Ji Hoon Yu, Sang-Hyun Min, Jongman Yoo

**Affiliations:** 1 Department of Medical Informatics, CHA University, Seongnam-si, Gyeonggi-do South Korea; 2 Department of Microbiology, CHA University, Seongnam-si, Gyeonggi-do South Korea; 3 Organoid Research Center, School of Medicine, CHA University, Seongnam-si, Gyeonggi-do South Korea; 4 Department of Internal Medicine, CHA Bundang Medical Center, CHA University, Seongnam-si, Gyeonggi-do South Korea; 5 Department of Physiology, School of Medicine, CHA University, Seongnam-si, Gyeonggi-do South Korea; 6 New Drug Development Center, Daegu-Gyeongbuk Medical Innovation Foundation, Dong-gu, Daegu South Korea

## Abstract

Sepsis is one of the most common clinical syndromes that causes death and disability. Although many studies have developed drugs for sepsis treatment, none have decreased the mortality rate. The aim of this study was to identify a novel treatment option for sepsis using the library of integrated network-based cellular signatures (LINCS) L1000 perturbation dataset based on an *in vitro* and *in vivo* sepsis model. Sepsis-related microarray studies of early-stage inflammatory processes in patients and innate immune cells were collected from the Gene Expression Omnibus (GEO) data repository and used for candidate drug selection based on the LINCS L1000 perturbation dataset. The anti-inflammatory effects of the selected candidate drugs were analyzed using activated macrophage cell lines. CGP-60474, an inhibitor of cyclin-dependent kinase, was the most potent drug. It alleviated tumor necrosis factor-α (TNF-α) and interleukin-6 (IL-6) in activated macrophages by downregulating the NF-κB activity, and it reduced the mortality rate in LPS induced endotoxemia mice. This study shows that CGP-60474 could be a potential therapeutic candidate to attenuate the endotoxemic process. Additionally, the virtual screening strategy using the LINCS L1000 perturbation dataset could be a cost and time effective tool in the early stages of drug development.

## Introduction

Sepsis is a systemic inflammatory syndrome caused by the activation of the innate immune system initiated by an acute microbial infection. It is the most common cause of mortality in critically ill patients ending in death in 30–50% of cases even in early treatment, and the majority of survivors develop long-term disabilities^1,2^. Epidemiologic studies have reported that the number of patients diagnosed with severe sepsis and septic shock is increasing^3^. Accordingly, the cost for sepsis treatment is quite high with more than 20 billion dollars spent annually in the United States^4,5^. Research on developing treatments for sepsis has been actively conducted worldwide over the past decade. Previously, Drotrecogin alfa was the only FDA approved sepsis treatment; however, it was withdrawn from the market due to its ineffectiveness^6^. Currently, there are no sepsis-specific therapeutics available in the market^7^. Previous development strategies for sepsis drugs have focused on specific anticytokine treatments. The rationale for such a strategy is that blocking important cytokines or related molecules such as toll-like receptor (TLR), interleukin-6 (IL-6), and tumor necrosis factor-α (TNF-α) could inhibit the progression of sepsis^8–10^. As one aspect of the inflammatory responses, a net procoagulant state due to sepsis promotes the mechanisms for dysfunctional endogenous anticoagulant and impaired fibrin removal by a suppressed fibrinolytic system^10^. However, drugs for such therapeutic targets have not been effective in stopping the progression of sepsis^7,11^, and no successful drugs for the specific treatment of sepsis have been developed^12,13^.

Recently, gene expression studies based on microarrays have suggested a ‘genomic storm’, which means that sepsis represents a complex response affecting more than 80% of cellular functions and pathways^9^. Establishing a therapeutic strategy based on gene expression in sepsis patients has been shown to be effective in promoting therapeutic outcomes^14^. This suggests that “-omics” level approaches will be required for the successful development of sepsis drugs.

By using a virtual screening of the connectivity map methodology based on the integration of the available gene expression data, it is possible to systematically discover effective therapeutic drugs^15,16^. By pairing drugs that correlate with abnormal gene expression in a disease, effective drug-disease pairs and new indications for currently approved drugs can be identified^17^. Using this approach, therapeutic candidates for various diseases such as dyslipidemia^18^, pain^19^, influenza^20^, lung cancer^21^, and renal cancer^22^ have been identified, and the methodological validity of the connectivity map has been confirmed as a promising technique for drug development and repositioning^16^.

Virtual screening using a connectivity map requires a gene expression profiling database of small molecules to be compared with the gene expression signatures of a disease. The library of integrated network-based cellular signatures (LINCS) L1000 dataset currently has over a million gene expression profiles in small molecule treated human cell lines. Candidates are predicted by comparing the LINCS L1000 perturbation signatures and the disease specific signatures extracted from the gene expression omnibus (GEO)^23^.

This study shows that CGP-60474, a drug identified by the virtual screening strategy using the LINCS L1000 perturbation dataset, attenuates the endotoxemic process in *in vitro* and *in vivo* models. The results of this study provide valuable insight on a drug development process that can normalize the expression pattern of sepsis using a connectivity map algorithm based on the GEO and LINCS L1000 databases. To confirm the therapeutic effectiveness of the candidate drugs, their therapeutic effects were also investigated in cellular and animal endotoxemic models.

## Results

### Initial identification of candidate drugs to treat sepsis by disease-drug associations

**Figure 1:**
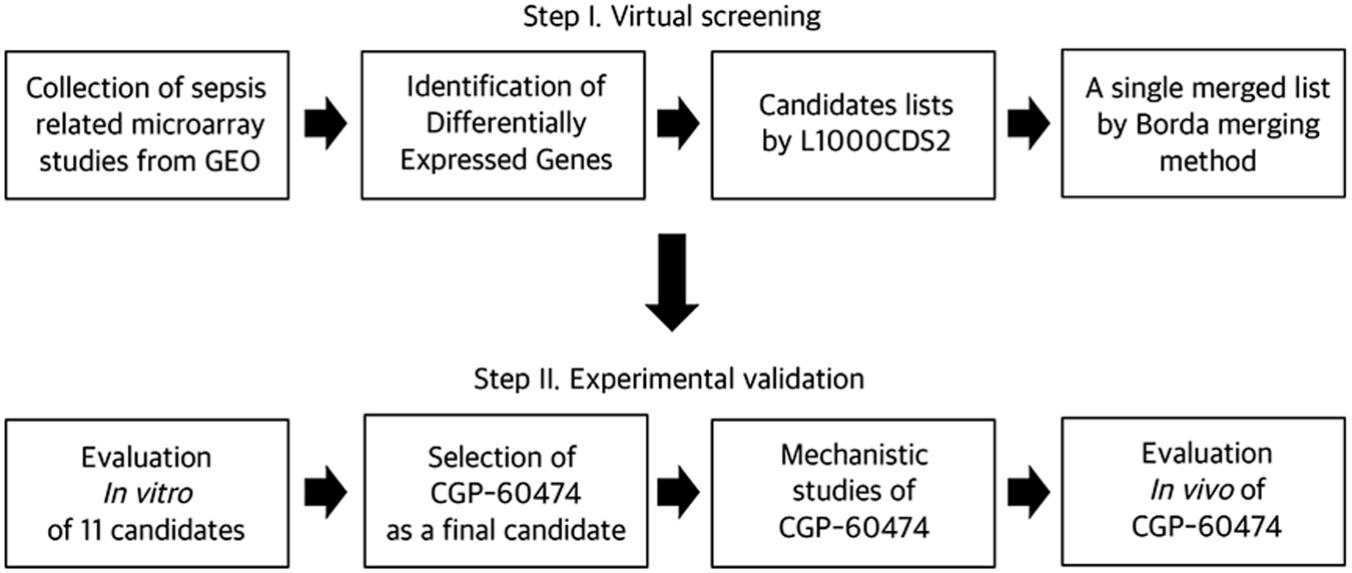
Experimental design. For the virtual screening for sepsis treatment, DEGs were extracted using sepsis-related microarrays from the GEO. DEGs were inputted into the L1000CDS^2^ to obtain candidate compounds, and they were scored and ranked. In the next step of experimental validation, some of the candidate drugs were shown to have therapeutic effects in cellular and animal models of endotoxemia. Additional information for DEGs was obtained through ontology, pathway, and PPI network analysis.

To screen the LINCS L100 perturbation database to identify candidate drugs for treating sepsis, we collected four sepsis-related microarray studies from the GEO data repository focusing on early-stage inflammatory processes and LPS-mediated systemic inflammation (Table 1 and Fig. 1). All differentially expressed gene symbols from the four microarray results were merged into 749 unique DEG symbols and used for the virtual screening (Fig. S1). Next, we searched the L1000CDS^2^ to prioritize small-molecules that were predicted to be either a reverse or mimic expression of the DEG signatures^23^. The L1000CDS^2^ calculates the pair-wise cosine distance between the directions of the disease-drug characteristics and provides ranked lists of scores for the candidate compounds. Then, we integrated five lists of small-molecules into a single ranked list with the Borda merging method with a base-score of 50 to include drugs that appear consistently and are high-ranked^24^. The scores reflect the rank of each drug and the consistency between different settings on the ranked lists (Table S2). Interestingly, among the top ranked drugs, dasatinib has been reported to have dual effects on polymicrobial sepsis [9]. Administration of I-BET increases the survival rate in the LPS endotoxemia and CLP-induced sepsis models^25^.

**Table 1:** Description of GEO datasets used in this study.

GEO accession number	Sample No. of Control	Sample No. of Experiment	Species	Experimental Design
GSE57065	82	25	Homo sapiens	Early and dynamic changes in gene expression in septic shock patients: a genome wide approach
GSE60290	3	2	Mus musculus	Interferon-gamma effect on lipopolysaccharide-activated bone marrow-derived macrophages
GDS5196	4	4	Mus musculus	Interferon-γ and lipopolysaccharide treatment effect on bone marrow derived macrophages
GDS2856	2	8	Homo sapiens	Peripheral blood-derived monocytes response to lipopolysaccharide: time course

Four sepsis-related microarray studies from the GEO data repository focusing on early-stage inflammatory processes and LPS-mediated systemic inflammation were used to identify candidate compounds by L1000CDS^2^.

We constructed a sepsis network from the perspective of protein-protein interactions to identify important hub genes for the disease and to better understand their effects on disease progression. For the topological analysis, the betweenness and the degree centrality for the genes are listed in Table S2. Our results show that the TNF signaling pathway, Toll like receptor-4 (TLR-4) cascade and Type I Interferon signaling are significant based on the pathway enrichment analysis (Fig. S2). From the perspective of the centrality of the PPI network, this result suggests that most of the interacting and bottleneck genes like NFKB1, SRC and MYC could have important roles in the pathogenesis of sepsis.

### Experimental validation of the candidate drugs in a cellular model of sepsis

To confirm that the virtual screening is useful, the efficacy of the top ranked 11 candidate drugs was verified *in vitro* (Table S2). Bone marrow derived macrophages (BMDM) and J774.1 cells, murine macrophage cell lines, were activated with lipoteichoic acid (LTA) as a gram positive bacterial endotoxin or lipopolysaccharide (LPS) as a gram negative bacterial endotoxin and then treated with each candidate drug^26,27^. TAK-242, previously known as a TLR-4 antagonist, was used as a positive control^28^. TNF-α and IL-6 are cytokines primarily produced by activated macrophages in response to microbial invasion and have an important role in the early phase of sepsis^8^. Because an extremely high level of IL-6 has been reported to be associated with the severity of sepsis and its levels are significantly decreased in the survivors of sepsis, IL-6 is a good prognostic marker for sepsis^29^. To evaluate the anti-inflammatory effects of each candidate drug, the TNF-α and IL-6 levels were evaluated in J774.1 cells activated by 100 ng/ml LPS (Fig. S3). The levels of secreted TNF-α and IL-6 were also investigated in BMDMs after co-treatment with 100 ng/ml LPS (Fig. S4A,B), pre-treatment with 100 ng/ml LPS (Fig. S4C,D), or co-treatment with 1 ug/ml LTA (Fig. S4E,F). Among the 11 drugs, dasatinib, geldanamycin, and NVP-AUY922 showed anti-inflammatory effects under various stimulating conditions in both the J774.1 cells and BMDMs.

To determine whether the decrease in cytokine release was due to a decrease in the number of viable cells due to cytotoxicity, the cytotoxicity of the 11 candidate drugs was also measured by the WST-1 activity to confirm their cell viability in the J774.1 cells and BMDMs. As a result, geldanamycin significantly decreased the cell viability at a concentration of 10 μM suggesting that the decrease in the TNF-α and IL-6 secretion is due to cell toxicity rather than an anti-inflammatory effect. Because dasatinib and NVP-AUY922 had a higher concentration for cell toxicity than for anti-inflammatory effects, cytokine release was reduced due to anti-inflammatory effects (Fig. S5). Altogether, two primary hits out of eleven drugs were found demonstrating that the virtual screening strategy using the L1000CDS^2^ is an effective method for identifying sepsis drugs.

### CGP-60474 inhibits the IL-6 level following LPS or Poly (I:C) administration *in vitro*

**Figure 2:**
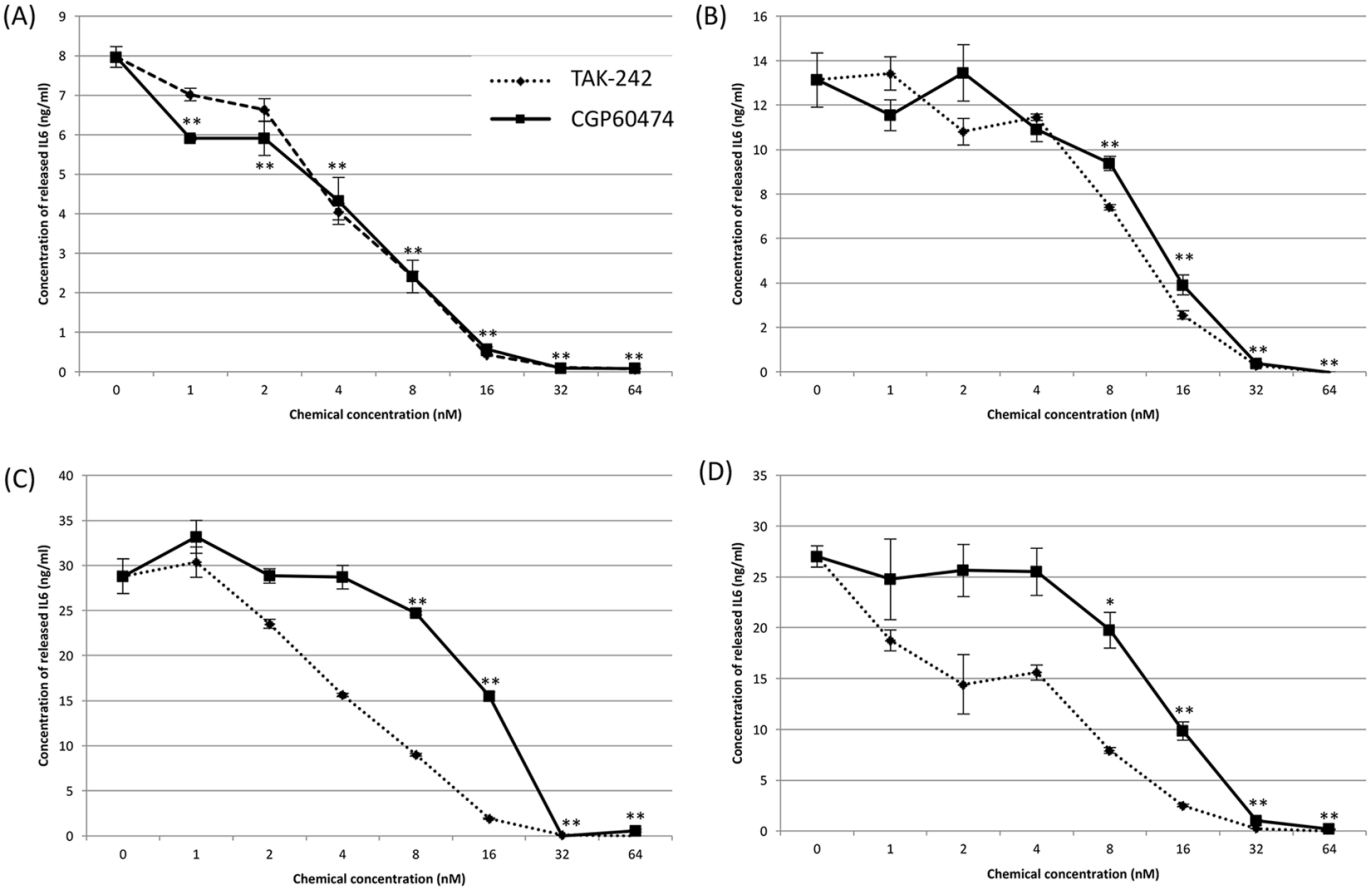
Inhibitory effects of CGP-604474 on IL-6 release following LPS or Poly (I:C) administration *in vitro*. (**A**) The concentration-dependent inhibition of IL-6 secretion when J774.1 cells were co-treated with 100 ng/ml LPS and CGP-60474. (**B**) The concentration-dependent inhibition effect on IL-6 secretion when J774.1 cells were treated with CGP-60474 at 30 min after treatment with 100 ng/ml LPS. The amount of IL-6 secretion decreased in a concentration-dependent manner by treatment with CGP-60474. (**C**) The concentration-dependent inhibition of IL-6 secretion when J774.1 cells were co-treated with 10 μg/ml poly(I:C) and CGP-60474. (**D**) The concentration-dependent inhibition effect on IL-6 secretion when J774.1 cells were treated with CGP-60474 at 30 min after treatment with 10 μg/ml poly(I:C). CGP-60474 successfully inhibited the IL6 secretion in J774.1 cells after poly(I:C) administration similar to the positive control drug TAK-242. Data are presented as the mean ± SEM; Experiment was independently repeated three times, **p < 0.01, *p < 0.05.

CGP-60474, which had the highest score in the virtual screening, is a potent inhibitor of cyclin-dependent kinase, which is known to be associated with controlling cell cycle transitions and other important cell functions^30^. To further analyze the anti-inflammatory effects of CGP-60474, we treated LPS stimulated J774.1 cells with CGP-60474, and the IL-6 secretion was measured in the cell culture supernatants. As a result, the amount of IL-6 secretion decreased in a concentration-dependent manner by the treatment with CGP-60474 (Fig. 2A,B). Similarly, IL-6 secretion was induced in J774.1 cells by treatment with polyinosinic-polycytidylic acid (poly(I:C)) as a ligand for cytosolic TLR3. CGP-60474 successfully inhibited the IL6 secretion in poly(I:C) treated J774.1 cells similar to the positive control drug TAK-242 (Fig. 2C,D).

**Figure 3:**
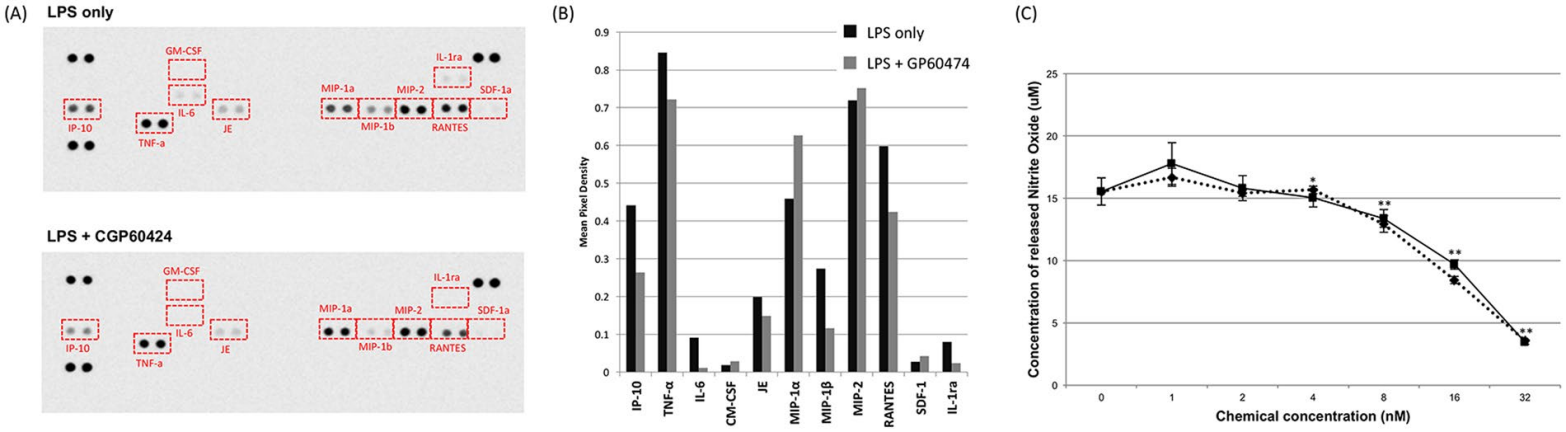
Profiling of cytokine changes by CGP-60474 in LPS treated J774.1 cells. 100 ng/ml LPS treated J774.1 cells were treated with vehicle or CGP-60474. The cultured supernatants collected 24 hours later were blotted for 40 cytokines simultaneously (**A**) and quantified (**B**). The GM-CSF and SDF-1, which are known to have important roles in the recovery of sepsis, were increased by the CGP-60474 treatment. (**C**) NO secretion by the LPS-stimulated J774.1 cells were reduced in a concentration dependent manner by the treatment with CGP-60474. Data are presented as the mean ± SEM; Experiment was independently repeated three times, **p < 0.01.

To determine whether CGP-60474 could change the secretion of other cytokines besides just IL-6, a cytokine profiling analysis was performed to observe 40 cytokines simultaneously. After LPS stimulated J774.1 cells were treated with CGP-60474, the pro-inflammatory cytokines IP-10, TNF-a, IL-6, MIP-1b and RANTES were decreased. GM-CSF^31^ and SDF-1^32^, which are known to have an important role directly or indirectly in the recovery of sepsis, were increased by the CGP-60474 treatment (Fig. 3A,B).

To determine whether the decrease in cytokine release was due to a decrease in cell number due to the cytotoxicity of the drug, the cytotoxicity of CGP-60474 was confirmed by the WST-1 assay in the J774.1 cells and BMDMs. Growth inhibition was observed at concentrations above 32 nM in both the J774.1 cells (Fig. S6A) and BMDMs (Fig. S6B). The cytotoxicity-initiating concentrations were higher than those with anti-inflammatory effects.

Taken together, our results consistently show that the CGP-60474 treatment in the endotoxemia cellular models regulates the excessive immune response in sepsis.

### Production of nitric oxide is inhibited by CGP-60474 in LPS-stimulated macrophages

In sepsis, cytokine-dependent induced nitric oxide synthase (iNOS) is activated, which results in excessive nitric oxide (NO) production. This results in cellular and organ dysfunction of the cardiovascular system and liver due to septic shock^33^. To investigate whether CGP-60474 inhibits the NO production of LPS-stimulated macrophages, LPS-stimulated J774.1 cells and BMDMs were incubated with various concentrations of CGP-60474. The secretion of NO was determined by the Griess assay at 24 hours after the treatment in the cell culture supernatant. In the J774.1 cells, NO secretion by the LPS-stimulation was reduced in a concentration dependent manner by the treatment with CGP-60474 (Fig. 3C). A similar result was also observed in the LPS-stimulated BMDMs (Fig. S7). These results support our hypothesis that the reduction of NO in activated macrophages by the treatment with CGP-60474 contributed, at least partly, to the anti-endotoxemic effects in sepsis.

### CGP-60474 inhibits NF-κB signaling following LPS or Poly (I:C) administration

**Figure 4:**
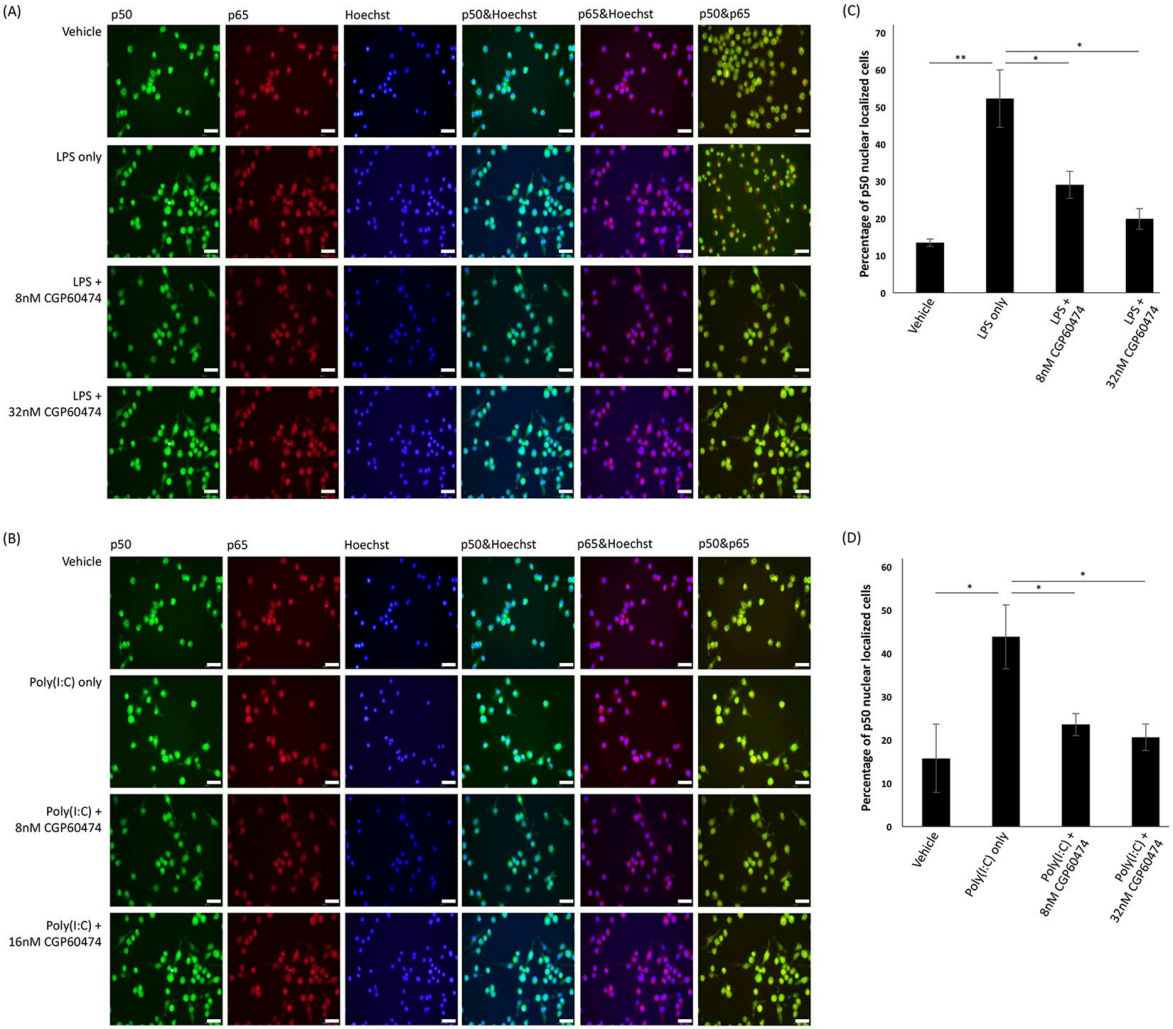
Inhibitory effects of CGP-604474 on NF-κB nuclear translocation following LPS or Poly (I:C) administration *in vitro*. (**A**) The expression of NF-κB p50 and p65 by treatment with CGP-60474 was examined in 100 ng/ml LPS treated J774.1 cells. (**B**) The expression of NF-κB p50 and p65 by treatment with CGP-60474 was examined in 10 μg/ml poly(I:C) treated J774.1 cells. (**C**,**D**) The results of NF-κB subunit p50 quantification show that both CGP-60474 treated LPS or poly(I:C) group significantly reduced p50 nuclear localized cells. Bars, 50 μm Data are presented as the mean ± SEM; Experiment was independently repeated three times, **p < 0.01, *p < 0.05.

Activation of TLR mediated NF-κB signaling induces nuclear translocation of the NF-κB p50 and p65 subunits. NF-κB signaling is involved in regulating the transcription of sepsis related immunomodulatory mediators associated with the onset of sepsis-triggering organ failure such as TNF-α, IL-6 and iNOS^34^. To investigate whether CGP-60474 inhibits NF-κB signaling, NF-κB p50 and p65 were visualized by immunocytochemistry following LPS or poly(I:C) administration in J774.1 cells (Fig. 4A,B). The CGP-60474 treated groups had a significant reduction in the nuclear translocation of the NF-κB p50 subunit compared to the control (LPS or poly(I:C) only) groups (Fig. 4C,D). Similar results were also observed in the LPS-stimulated BMDMs (Fig. S8).

This result suggests that the CGP-60474 treatment was effective in reversing the NF-κB translocation due to the endotoxin stimulation, which is consistent with our observation.

### CGP-60474 inhibits the IL-6 level and increases the survival rate in the LPS endotoxemia model

**Figure 5:**
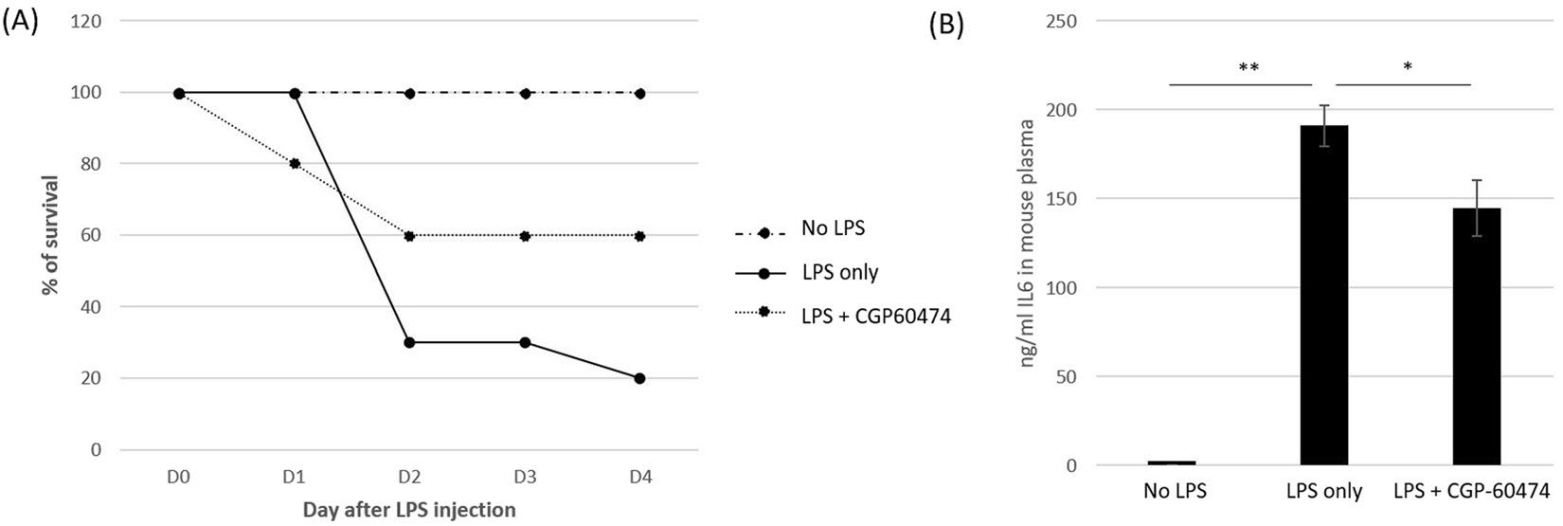
Therapeutic effects of CGP-60474 on the LPS endotoxemia model. (**A**) Mice were injected with 10 mg/kg LPS intraperitoneally and CGP-60474 was administered 30 minutes later. The survival rate was observed for 4 days. The mice treated with CGP-60474 had higher survival rate than the mice treated with the vehicle after the GCP-60474 treatment. (**B**) Plasma IL-6 concentrations were measured 2 hours after LPS administration. The level of IL-6 decreased in the mice group treated with the GCP-60474. Data are presented as the mean ± SEM; Experiment was independently repeated three times, total n = 10 mice per group, **p < 0.01, *p < 0.05.

LPS induced endotoxemia induces overproduction of pro-inflammatory cytokines and the accumulation of neutrophils in the lung with other clinical endotoxic shock-like symptoms such as systemic arterial hypotension, lactic acidosis, or impaired myocardial contractility culminating in multi-organ failure and death from endotoxic shock^35^. CGP-60474 or vehicle was intraperitoneally injected into a LPS endotoxemia mouse model. Notably, the mice group treated with CGP-60474 had a higher survival rate than the mice group treated with the vehicle after the GCP-60474 treatment (Fig. 5A). Furthermore, a decreased plasma level of IL-6 was also observed compared with the mice treated with the vehicle (Fig. 5B).

These results show that CGP-60474 has anti-endotoxemic effects in an animal model as well as in cellular models suggesting that CGP-60474 eventually suppresses the progression of sepsis.

## Discussion

Sepsis is initiated by pathogenic microorganisms; however, heterogeneous syndrome as a result of a pathogen and host interactions provoke a complex network of inflammatory cascades in multiple organs^36^. From this perspective, recent studies have investigated and identified genetic variations associated with sepsis. Pachot *et al*. investigated a set of genes differentially regulated between non-survivors and survivors of sepsis^37^. Several studies on children with septic shock have reported different gene expressions relative to normal children which reported that specific genetic polymorphisms are broadly involved in inflammation and immunity and are linked with susceptibility to sepsis^38,39^. We also thought that any treatment of sepsis only blocking single molecules of specific anticytokines would not be effective and that a systematic approach would be needed. This study elucidated efficacious drugs for the treatment of sepsis using a virtual screening strategy based on the LINCS L1000 perturbation database. Then, we verified the efficacy of the candidate drugs in a cellular and animal model of endotoxemia. To evaluate the therapeutic potential of the top ranked drug CGP-60474 in the virtual screening, it is necessary to examine the effect of CGP-60474 when delivered at multiple phases of the endotoxin stimulation. Furthermore, a mouse model assessment is essential to understand the therapeutic effects of CGP-60474 in sepsis.

In this study, we examined whether CGP-60474 in the endotoxin stimulation relieved the excessive innate immune responses that result in tissue and organ failures. When the endotoxin-activated macrophages were treated with CGP-60474, a concentration-dependent inhibition of inflammatory cytokines and the NF-κB signaling pathway was evident. In addition, the mice treated with CGP-60474 had a higher survival rate, and the plasma IL-6 was decreased in the mice group treated with the GCP-60474. These results suggest that CGP-60474 could be a therapeutic agent that attenuates the endotoxemia-induced systemic state.

CGP-60474 is a small chemical molecule and potent inhibitor of cyclin-dependent kinase (CDK) 1 and CDK2. Its chemical name is 3-(4-2(-3-chlorophenylamino)pyridimin-4-yl)pyridine-2-ylamino)propanol^30^. CDKs are known to control cell cycle transitions and other important cell functions like transcription, and there have been many studies that have developed CDK inhibitors as anti-cancer agents^40^. For example, Camilla *et al*. used a high-throughput cellular screen of a diverse chemical library to identify drugs for small cell lung cancer (SCLC)^41^. They screened small molecules that suppress the SCLC cell growth, and CGP-60474 was one of the top-ranked inhibitors of transcription and cell cycle function. p21 inhibits cell-cycle progression by targeting cyclin dependent kinase 2, and a previous study showed that LPS-stimulated p21-deficient macrophages had an increased activity of the transcription factor NF-κB^42^. This result is in agreement with our results which show the inhibitory effects of CGP-60474 on NF-κB nuclear translocation. In addition, a recent study on the CDK inhibitor AT519 also suggested that this CDK inhibitor accelerates neutrophil apoptosis in sepsis-related acute respiratory distress syndrome^43^. The current concept of sepsis is life-threatening organ dysfunction caused by a dysregulated host response [28752002], and NF-κB signaling is known to be an one of the important events in the pathophysiology of sepsis [23975688]. Further studies on the mechanisms of the CDK pathways and the therapeutic effects of CDK inhibitors in sepsis-related signaling abnormalities could be helpful in designing therapeutic strategies for this drug on the treatment of sepsis.

At present, the results of this study show that our method is a reliable and effective; moreover, among the candidates positively predicted by the bioinformatics-based pipeline that identifies novel drug candidates by combining the disease transcriptome database from GEO and the small molecular perturbation database from the LINCS L1000, the discovered compounds were effectively evaluated by experimental validation. Furthermore, additional disease network analysis and pathway enrichment analysis can provide a valuable global perspective and clues to biomedical researchers on the complex genetic links of disease genes and on the data interpretation of the mechanisms of repurposed drugs. However, one drawback of network approaches is a tendency for them to be somewhat *ad hoc* in nature, for example, no existing clear threshold values and publication biases. For further network-based research, well-defined designs and robust methods to reduce these biases and to increase the reproducibility for the disease network construction are needed.

Taken together, our results show that CGP-60474, a top ranked drug from our virtual screening, normalizes the endotoxin mediated immune dysfunctions and improves the survival rate in an animal model. As a consequence, the screening strategy by disease-drug association through public data sources will hopefully provide a method to discovery novel candidate drugs.

## Materials and Methods

### LINCS L1000 CDS^2^ Query

Microarray datasets for sepsis-related studies were identified in the GEO. Each dataset of the four sepsis-related microarray studies had RMA normalized intensities. Gene filtering was done with the genefilter R package^44^ to increase the detection power and to match the probe IDs to the gene symbol names. The probe IDs that did not have a matched symbol name were discarded, and duplicated symbol names with multiple probe IDs had the highest-IQR probe for each gene. Next, we analyzed the expression sets using Limma^45^ to select only statistically significant DEGs with different settings (adj. p-value < 0.05, Table S1). Limma is an R/Bioconductor software package that provides an integrated solution for analyzing data from gene expression experiments. Mouse gene symbols from Mus musculus were converted to human orthologous genes using the biomaRt R package^46^. A state-of-the-art multivariate approach called the Characteristic Direction (CD) method was used to identify disease signatures for each dataset^47^. The CD method is based on linear discriminant analysis (LDA) and characterizes differential expression to a single geometric direction of the disease-control separating hyper-plane. Multivariate methods may be more appropriate for transcriptional profiling regarding the dependencies between the expression levels of genes. The web-based search engine L1000CDS^2^ provides signatures for thousands of L1000 small-molecules and their combinations with the predicted target information. We derived the PPI network from 749 genes from previous preprocessing and estimated the basic topological measures of centrality. We first mapped the genes to STRINGdb^48^ (score threshold = 800), a database of known and predicted PPIs, and visualized the genes with the Cytoscape Application. The degrees of node and betweenness centrality were measured.

### Animals

This study was approved and conducted according to the regulations and guidelines of the CHA University Institutional Animal Care and Use Committee. All efforts were made to minimize animal suffering and to reduce the number of animals used. Male C57Bl/6 mice were obtained from the Orient Bio (Seongnam, Korea) and maintained in the animal facility of CHA University under 12-h light/dark cycle with food and water available *ad libitum*.

### Chemicals

OSI-930, Amuvatinb, NVP-AUY922, JNK-IN-5A, Geldanamycin, BI-2536, ZSTK-474, XMD8-92, Vorinostat, Dastatinib, Linifanib and Wortmannin were purchased from Targetmol (Boston, MA), TAK-242 from EMD Millipore (Billerica, MA), and CGP-60474 from MedchemExpress (Princeton, NJ).

### Preparation of bone marrow-derived macrophages (BMDMs)

For BMDM preparation, bone marrow cells were collected from the mouse femur and tibia, and differentiated into macrophages by incubation^49^. Briefly, mouse bones of femur and tibia were washed thoroughly and broken. The supernatant (Dulbecco’s phosphate-buffered saline, DPBS) was collected and filtered through cell strainer. The cell pellets were then embedded in red blood cell (RBC) lysis buffer for maximum 2 minutes. The pellets were washed and cultured in Petri dish for up to 7 days by adding DMEM (High glucose with 1% Penicillin/streptomycin and 10% Fetal bovine serum).

### Cytokine profiling

To simultaneously detect the changes of 40 cytokines, conditioned medium was collected at 24 hours after the compound treatment, and Proteome Profiler Array Mouse Cytokine Array (R&D Systems, Minneapolis, MN) was used. The supernatant of cells was collected after removing particles by centrifugation. The supernatant samples were embedded in block buffer and moved to a rocking platform shaker at room temperature for 60 min. Antibody cocktail from the kit covered the samples for overnight at 4 °C. Next day, the samples were washed and covered with Streptavidin-HRP as the following step. Other steps were followed by manufacturer’s guide.

### Quantitation of nitric oxide

To measure nitric oxide secretion, conditioned medium was collected at 24 h after the compound treatment. The amount of nitrite was measured with the Griess reaction assay (Thermo Fisher Scientific, Rockford, IL) following the manufacturer’s instructions. In brief, measurement of nitric oxide was performed through following steps. For preparation of Griess Reagent, equal volumes of N-(1-naphthyl) ethylenediamine (Component A) and sulfanilic acid (Compound B) were mixed together to form the Griess Reagent. For spectrophotometer assay, mixed 100 μl of Griess Reagent, 300 μl of the nitrite-containing sample, and 2.6 ml of deionized water were incubated at room temperature for 30 minutes. A photometric reference sample was prepared by mixing 100 μl of Griess Reagent and 2.9 ml of deionized water measured at 548 nm wavelengths. For microplate assay, 20 μl of Griess Reagent, 150 μl of the nitrite-containing sample, and 130 μl of deionized water were mixed and incubated at room temperature for 30 minutes. A photometric reference sample was mixed with 20 μl of Griess Reagent and 280 μl of deionized water. Experiment was independently repeated three times.

### Endotoxemic model and administration of CGP-60474 in mice

This study was approved and conducted according to the regulations and guidelines of the CHA University Institutional Animal Care and Use Committee. C57Bl/6 mice were injected with 10 mg/kg Lipopolysaccharide (LPS) from *Escherichia coli* O111:B4 (Sigma-Aldrich, St. Louis, MO) intraperitoneally. CGP-60474 was injected at 10 mg/kg at 30 minutes after LPS injection. Two hours after LPS injection, blood was collected from the retro-orbital plexus to obtain plasma. Experiment was independently repeated three times and used total 10 mice.

### Enzyme-linked immunosorbent assay (ELISA)

To quantify secreted IL-6 and TNF-α in cell culture supernatants, conditioned medium was collected at 24 hours after the compound treatment. The amount of IL-6 and TNF-α protein was detected and measured with ELISA (Biolegend, San Diego, CA) following the manufacturer’s instructions. To measure the IL-6 level in the mouse model, obtained plasma was used to perform ELISA.

### WST assay

The cells and medium from each group were used for cell viability with the EZ-Cytox assay kit (Daeil Lab Service, Seoul, Republic of Korea) by adding tetrazolium salt to each group. The samples were then incubated for 3 hours within dark environment. After 3 hours later, the medium from each sample was collected and moved to 96-well plate for measurement. A wavelength of 450 nm absorbance was measured by VersaMax ELISA Microplate Reader (Molecular Devices, CA, USA). Experiment was independently repeated three times.

### Statistical analysis

Differences between two groups were assessed by the Student’s t-test. Three or more groups were compared with one-way ANOVA followed by the Student-Newman-Keuls post hoc test. Differences were considered statistically significant at *p* < 0.05.

## Electronic supplementary material

Supplementary figures and tables
