# Supplementary material for: LINCS L1000 dataset-based repositioning of CGP-60474 as a highly potent anti-endotoxemic agent
Source: Sci Rep. 2018 Oct 8;8:14969. doi: 10.1038/s41598-018-33039-0 (PMC6175892; doi:10.1038/s41598-018-33039-0)
Supplement: Supplementary file 1 — Supplementary figures and tables [file 41598_2018_33039_MOESM1_ESM.docx]

**­­­­­­LINCS L1000 dataset-based repositioning of CGP-60474 as a highly potent anti-endotoxemic agent**

Hyun-Wook Han^a,*^, Soojung Hahn^b,c,*^, Hye Yun Jeong^c,d,*^, Joo-Hyun Jee^b,c^, Myoung-Ok Nam^b,c^, Han Kyung Kim ^b,c^, Dong Hyeon Lee^c,e^, So-Young Lee^d^, Dong Kyu Choi^f^ , Ji Hoon Yu^f^ , Sang-Hyun Min^f,2^, Jongman Yoo^b,c,2^

**Authors’ affiliation:**

^a^ Department of Medical Informatics, School of Medicine, CHA University, Seongnam-si, Gyeonggi-do, South Korea

^b^ Department of Microbiology and ^c^ Organoid Research Center, School of Medicine, CHA University, Seongnam-si, Gyeonggi-do, South Korea

^d^ Department of Internal Medicine, CHA Bundang Medical Center, CHA University, Seongnam-si, Gyeonggi-do, South Korea.

^e^ Department of Physiology, School of Medicine, CHA University, Seongnam-si, Gyeonggi-do, South Korea

^f^ New Drug Development Center, Daegu-Gyeongbuk Medical Innovation Foundation, Dong-gu, Daegu-si, South Korea

^*^These authors contributed equally to this work.

^2^ To whom correspondence should be addressed.

**Supplementary Information**

**SI Figures**

**
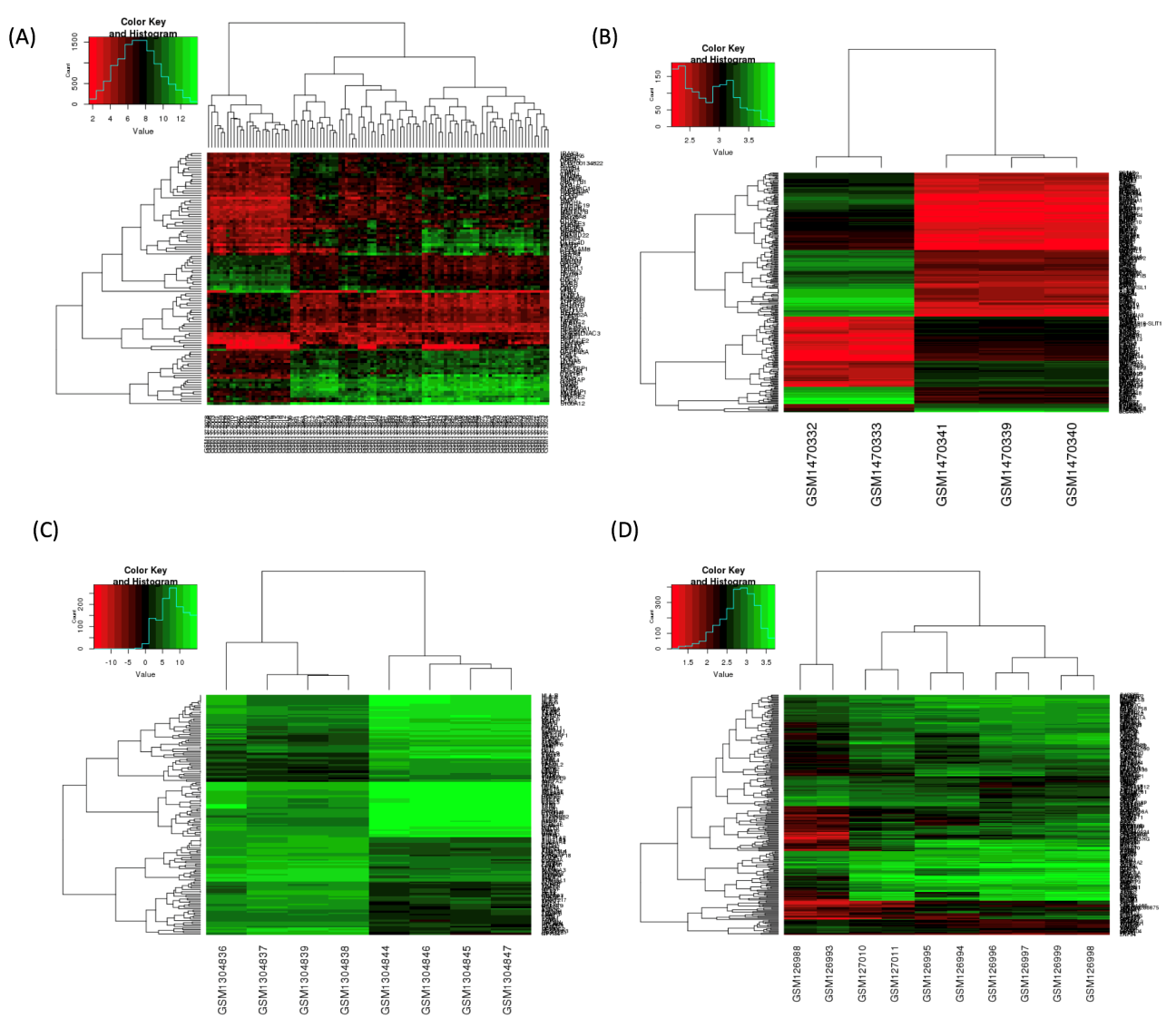
**

**Fig S1. Clustered heat map for sepsis related transcriptomes.**

A heat map was generated for each dataset using the parameters given in Supplementary Table 1. (A) GSE57065, (B) GSE60290, (c) GDS6196, and (D) GDS2856

**
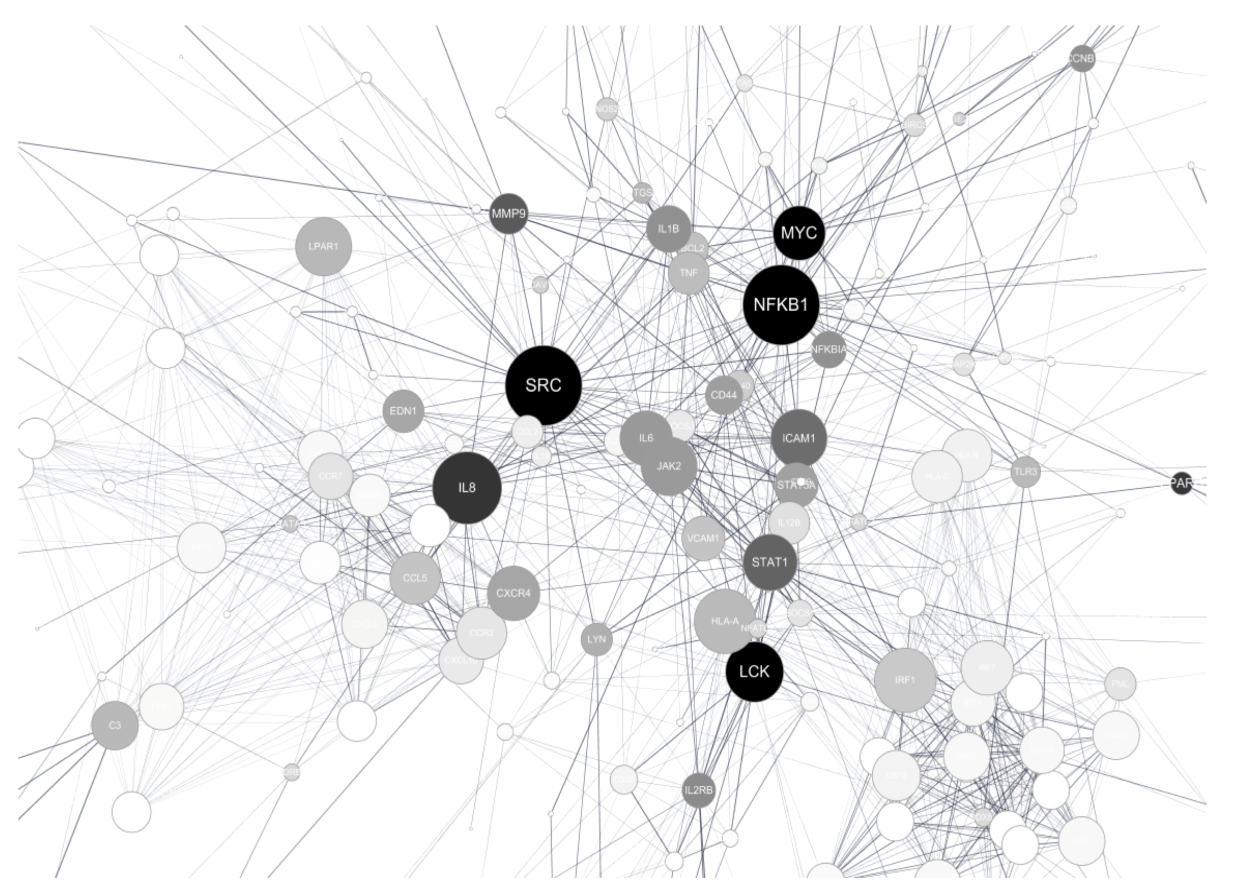
**

**Fig S2. PPI network visualization for sepsis-regulated genes from STRINGdb.**

PPI network was visualized using STRINGdb for sepsis-related DEGs. Node size represents degrees; node color gradient represents betweenness centrality.

**
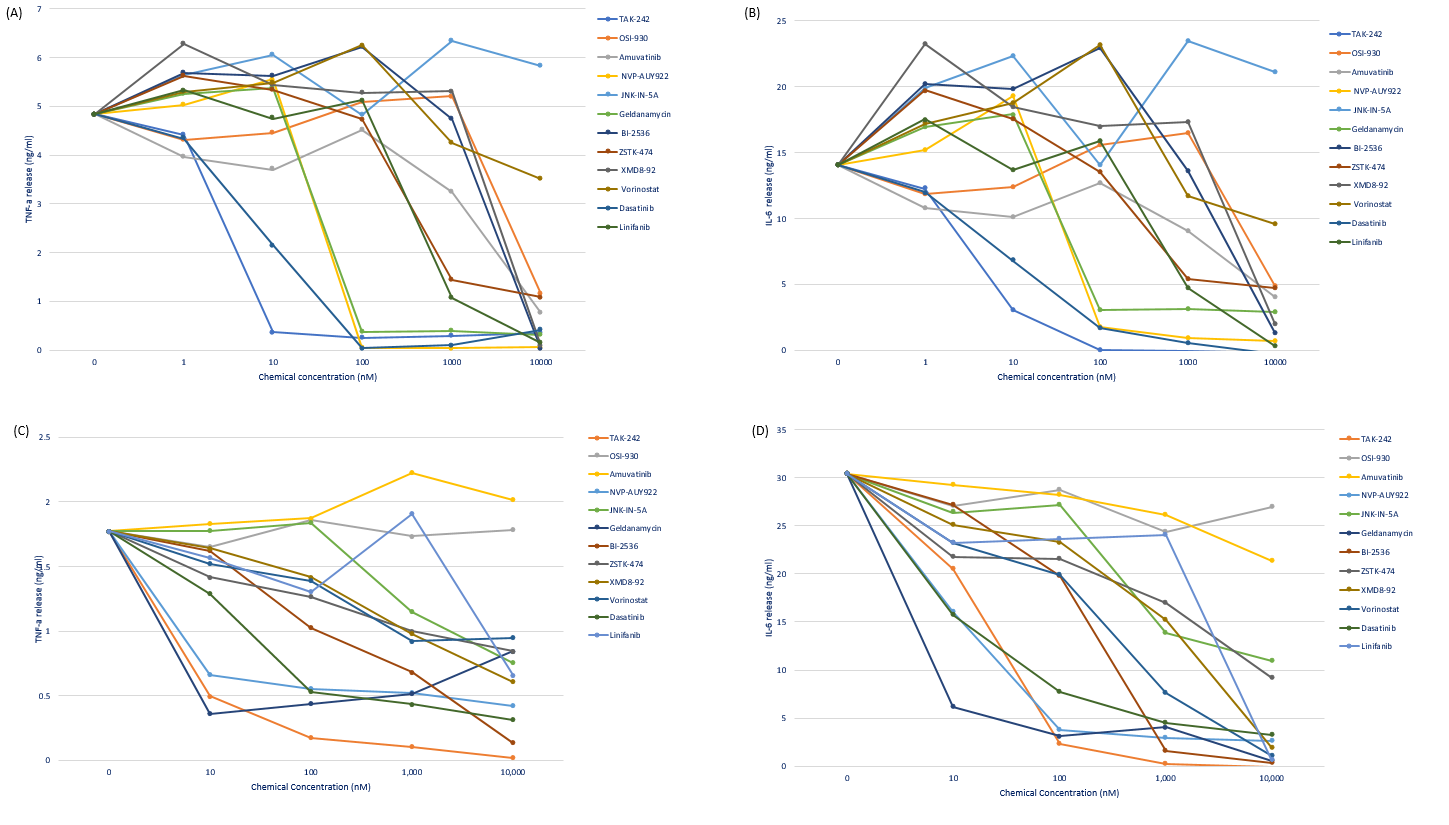
**

**Figure S3. Inhibitory effects of the 11 candidates on pro-inflammatory cytokine**

**release following LPS administration in J774.1 cells**

Concentration-dependent inhibition of TNF-α (A) or IL-6 (B) secretion when J774.1 cells were co-treated with 100 ng/ml LPS and the 11 candidates. The concentration-dependent inhibition effect on TNF-α (C) or IL-6 (D) secretion when J774.1 cells were treated with the 11 candidates at 30 min after the treatment with 100 ng/ml LPS. Data are presented as the mean ± SEM; n=3 samples per group


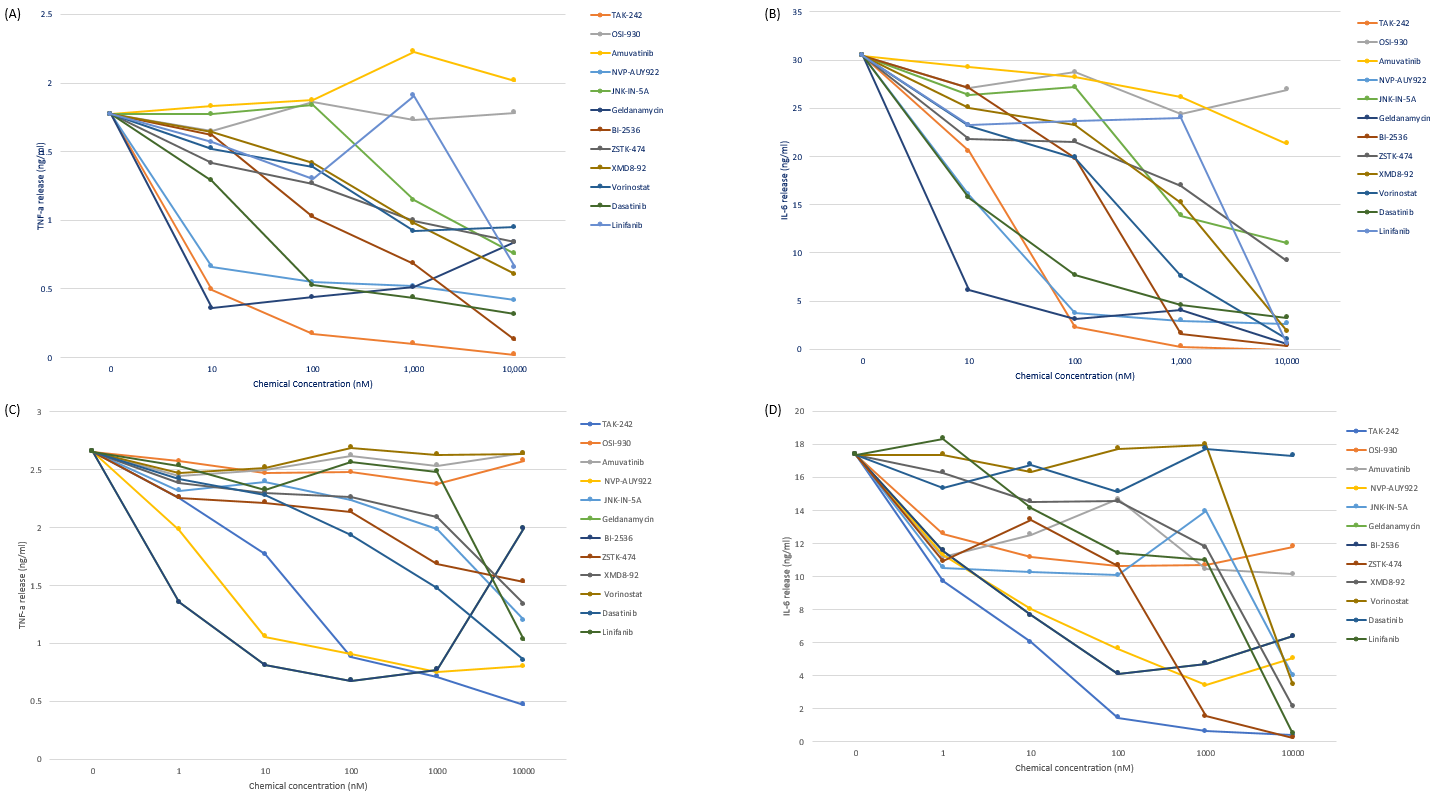


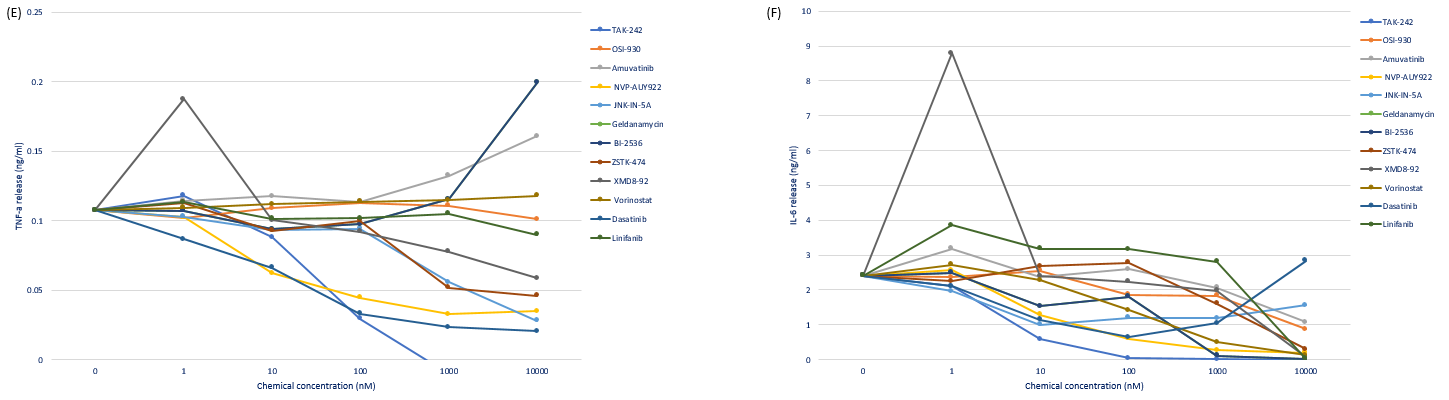


**Fig S4.** **Inhibitory effects of the 11 candidates on pro-inflammatory cytokine release following LPS administration in BMDMs**

Concentration-dependent inhibition of TNF-α (A) or IL-6 (B) secretion when BMDMs were co-treated with 100 ng/ml LPS and the 11 candidates. The concentration-dependent inhibition effect on TNF-α (C) or IL-6 (D) secretion when BMDMs were treated with the 11 candidates at 30 min after the treatment with 100 ng/ml LPS. Concentration-dependent inhibition of TNF-α (E) or IL-6 (F) secretion when BMDMs were co-treated with 1 μg/ml LTA and the 11 candidates. Data are presented as the mean ± SEM; n=3 samples per group


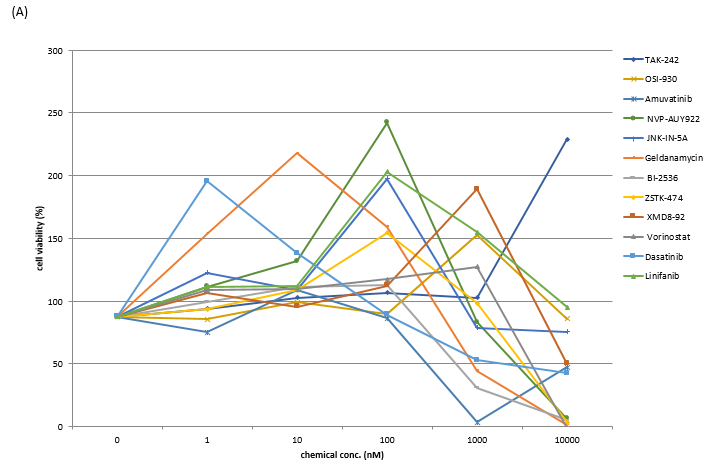

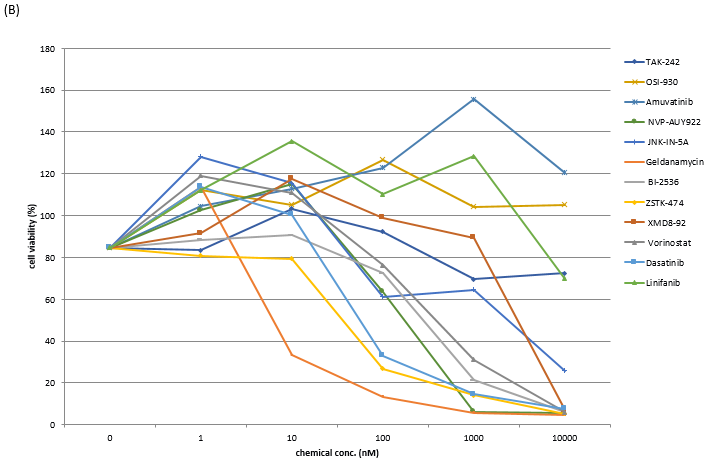


**Fig S5. Cytotoxicity test in J774.1 cells and BMDMs.**

The cytotoxicity of the 11 candidates was evaluated by the WST-1 assay in J774.1 cells (A) or BMDMs (B). Data are presented as the mean ± SEM; n=3 samples per group

**
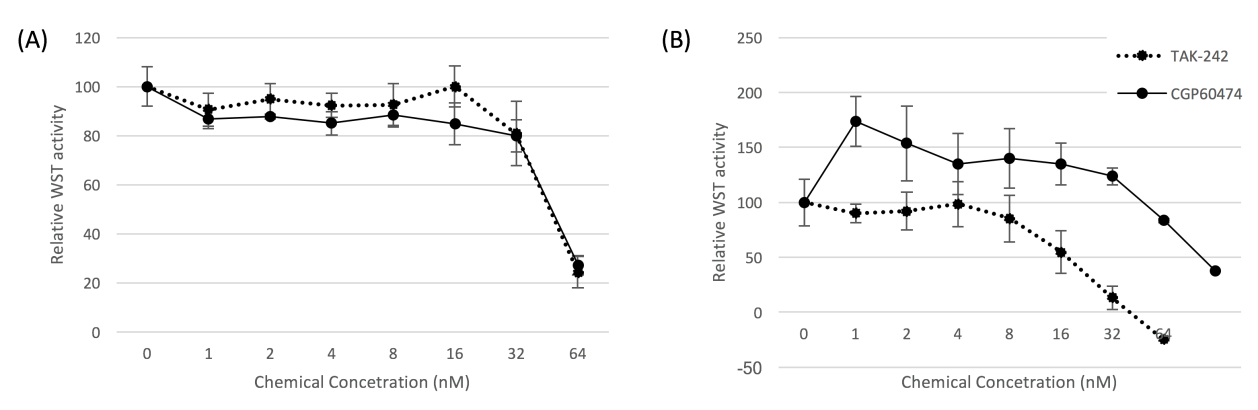
**

**Fig S6. Evaluation of cytotoxicity of CGP-60474 *in vitro***

The cytotoxicity of CGP-60474 was evaluated by the WST-1 assay in J774.1 cells (A)

and the growth inhibition was observed at concentrations above 32 nM in both the

J774.1 cells and BMDMs. J774.1 cells. Cytotoxicity-initiating concentrations were

higher than those with anti-inflammatory effects. Data are presented as the mean ±

SEM; n=3 samples per group.

**
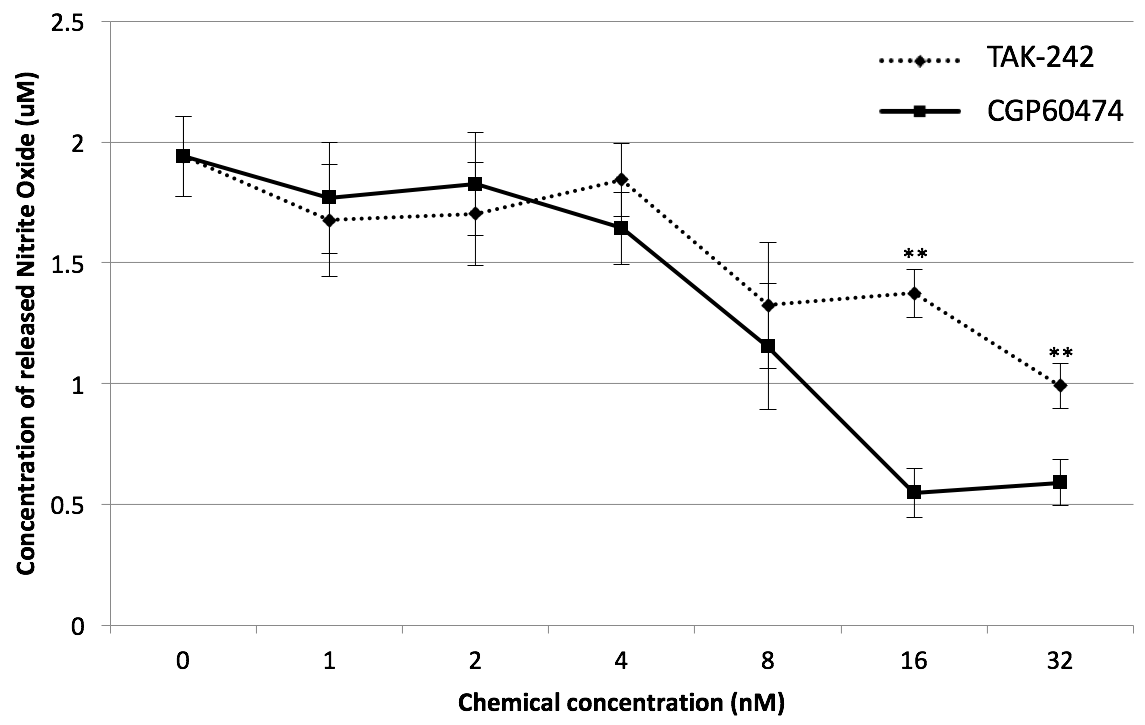
**

**Fig S7. Inhibitory effects of CGP-60474 on Nitric Oxide release in BMDMs**

NO secretion by the LPS-stimulated BMDMs were reduced in a concentration dependent manner by the treatment with CGP-60474. Data are presented as the mean ± SEM; n=3 samples per bar, **p < 0.01.


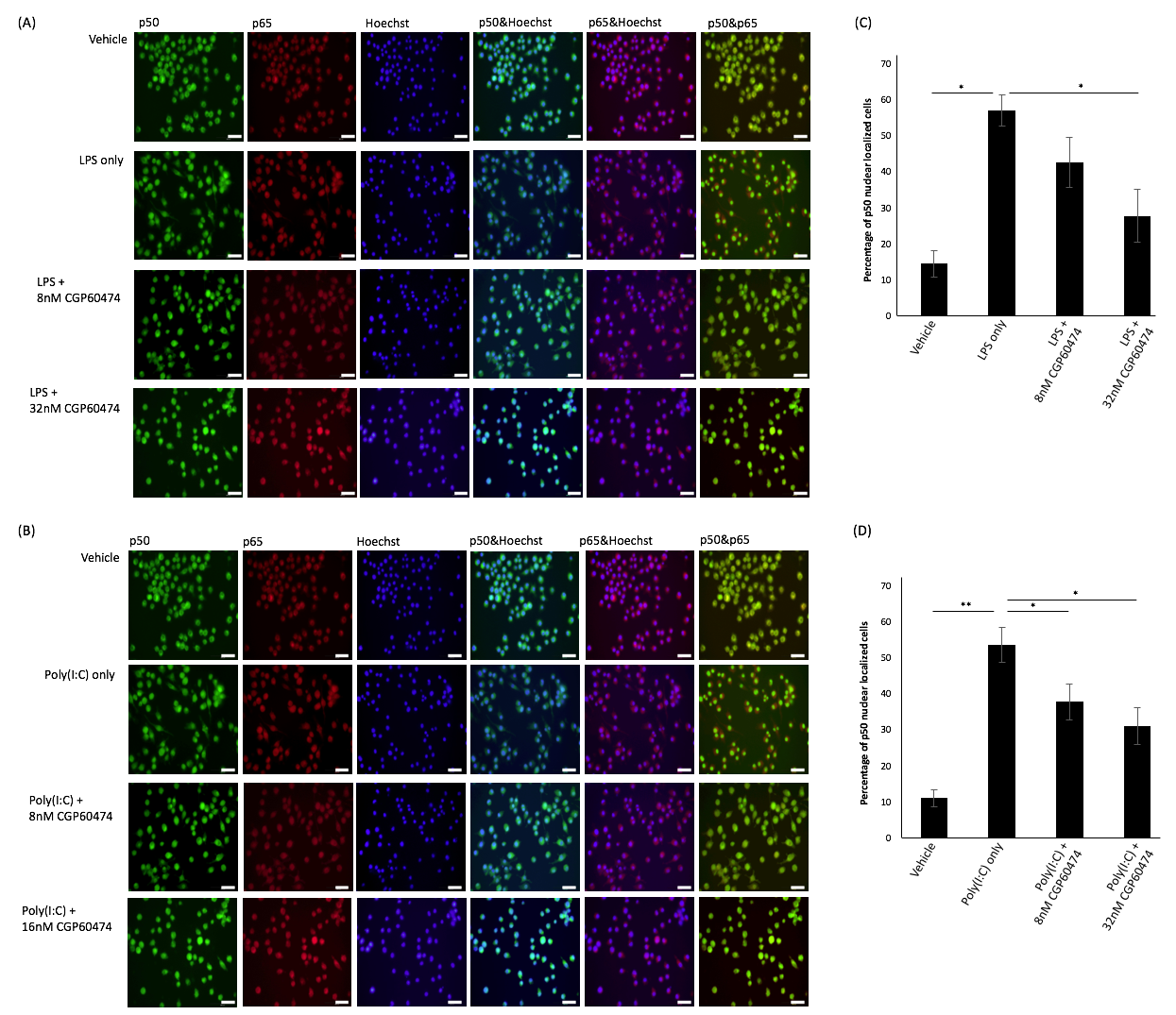


**Fig S8. Inhibitory effects of CGP-604474 on NF-kB nuclear translocation following LPS or Poly (I:C) administration in BMDMs**

(A) The expression of NF-kB p50 and p65 by treatment with CGP-60474 was examined in 100 ng/ml LPS treated BMDMs, and (B) The expression of NF-kB p50 and p65 by treatment with CGP-60474 was examined in 10 μg/ml poly(I:C) LPS treated BMDMs. Bars, 50 μm

**
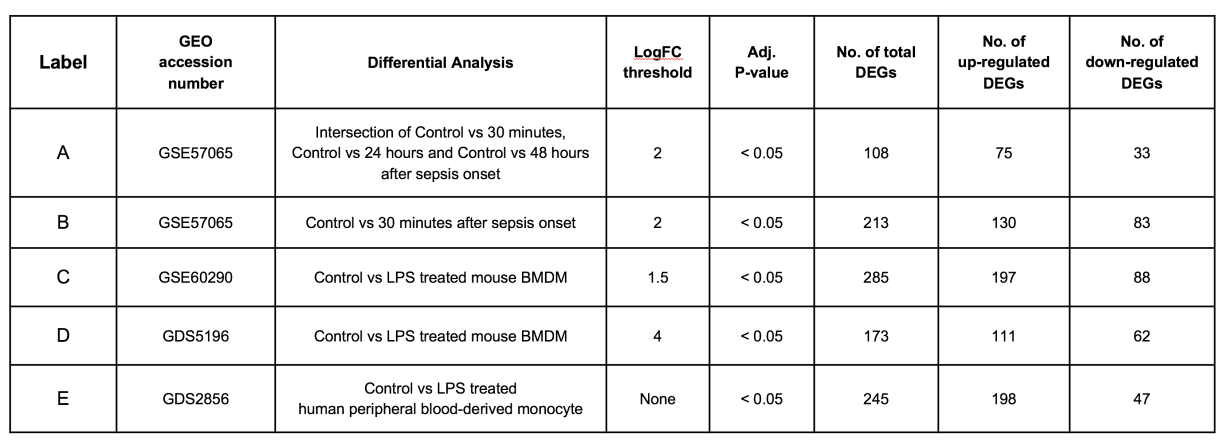
**

**Table S1. Dataset settings for preprocessing**

The four microarray datasets associated with sepsis obtained from the GEO were processed under five conditions to extract the DEGs. (A) GSE57065 was analyzed by microarray data from peripheral WBC of sepsis patients at 30 minutes, 24 hours, and 48 hours after sepsis onset. DEG was calculated for three time points, and then, we selected DEGs overlapping all three points. (B) GSE57065 dataset was used to select genes that changed at 30 minutes after sepsis onset. (C) GSE60290 dataset was used to select the genes that changed when mouse BMDMs were treated with LPS. (D) GDS5196 dataset was used to select the genes that changed when mouse BMDMs were treated with LPS. (E) GDS2856 dataset was used to select the genes that changed when human peripheral blood-derive monocytes were treated with LPS.

**
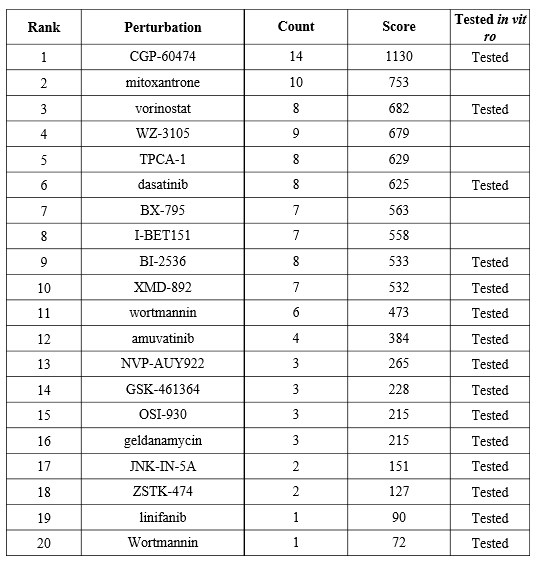
**

**Table S2. Scored List of perturbation by borda scores**

The scores reflect the rank of each drug and the consistency between different settings on the ranked lists.

**
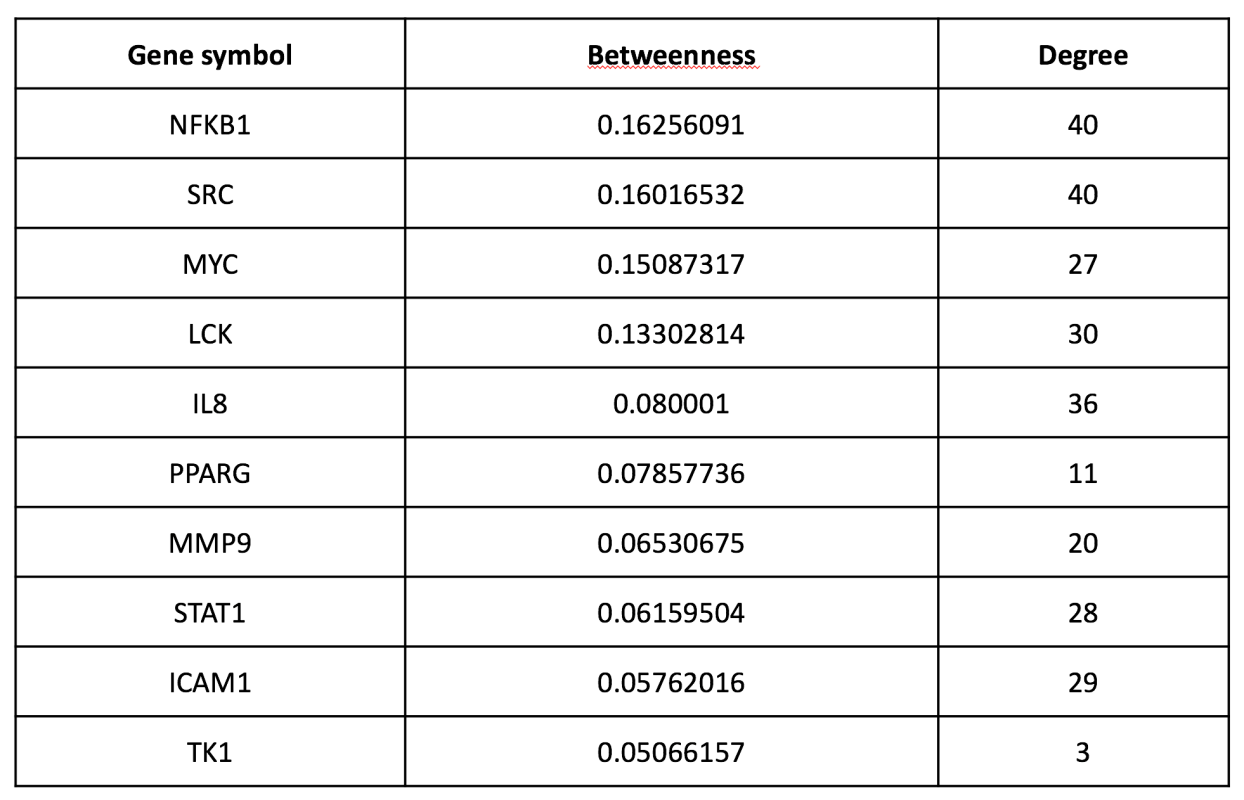
**

**Table S3. Top 10 genes by betweenness centrality**

In the PPI network derived from the sepsis DEGs, the top 10 genes with the highest degree of centrality were selected based on topological analysis. Most are known to be associated with sepsis.
